# DynaFusion-SLAM: Multi-Sensor Fusion and Dynamic Optimization of Autonomous Navigation Algorithms for Pasture-Pushing Robot

**DOI:** 10.3390/s25113395

**Published:** 2025-05-28

**Authors:** Zhiwei Liu, Jiandong Fang, Yudong Zhao

**Affiliations:** 1 College of Information Engineering, Inner Mongolia University of Technology, Hohhot 010080, China; 20231800102@imut.edu.cn; 2 Inner Mongolia Key Laboratory of Intelligent Perception and System Engineering, Hohhot 010080, China; zhaoyvdong@163.com; 3 Inner Mongolia Synergy Innovation Center of Perception Technology in Intelligent Agriculture and Animal Husbandry, Hohhot 010080, China

**Keywords:** pasturepushing, crawler robot, Cartographer, RTAB-Map algorithm, multi-sensor fusion, autonomous navigation

## Abstract

Aiming to address the problems of fewer related studies on autonomous navigation algorithms based on multi-sensor fusion in complex scenarios in pastures, lower degrees of fusion, and insufficient cruising accuracy of the operation path in complex outdoor environments, a multimodal autonomous navigation system is proposed based on a loosely coupled architecture of Cartographer–RTAB-Map (real-time appearance-based mapping). Through laser-vision inertial guidance multi-sensor data fusion, the system achieves high-precision mapping and robust path planning in complex scenes. First, comparing the mainstream laser SLAM algorithms (Hector/Gmapping/Cartographer) through simulation experiments, Cartographer is found to have a significant memory efficiency advantage in large-scale scenarios and is thus chosen as the front-end odometer. Secondly, a two-way position optimization mechanism is innovatively designed: (1) When building the map, Cartographer processes the laser with IMU and odometer data to generate mileage estimations, which provide positioning compensation for RTAB-Map. (2) RTAB-Map fuses the depth camera point cloud and laser data, corrects the global position through visual closed-loop detection, and then uses 2D localization to construct a bimodal environment representation containing a 2D raster map and a 3D point cloud, achieving a complete description of the simulated ranch environment and material morphology and constructing a framework for the navigation algorithm of the pushing robot based on the two types of fused data. During navigation, the combination of RTAB-Map’s global localization and AMCL’s local localization is used to generate a smoother and robust positional attitude by fusing IMU and odometer data through the EKF algorithm. Global path planning is performed using Dijkstra’s algorithm and combined with the TEB (Timed Elastic Band) algorithm for local path planning. Finally, experimental validation is performed in a laboratory-simulated pasture environment. The results indicate that when the RTAB-Map algorithm fuses with the multi-source odometry, its performance is significantly improved in the laboratory-simulated ranch scenario, the maximum absolute value of the error of the map measurement size is narrowed from 24.908 cm to 4.456 cm, the maximum absolute value of the relative error is reduced from 6.227% to 2.025%, and the absolute value of the error at each location is significantly reduced. At the same time, the introduction of multi-source mileage fusion can effectively avoid the phenomenon of large-scale offset or drift in the process of map construction. On this basis, the robot constructs a fusion map containing a simulated pasture environment and material patterns. In the navigation accuracy test experiments, our proposed method reduces the root mean square error (RMSE) coefficient by 1.7% and Std by 2.7% compared with that of RTAB-MAP. The RMSE is reduced by 26.7% and Std by 22.8% compared to that of the AMCL algorithm. On this basis, the robot successfully traverses the six preset points, and the measured X and Y directions and the overall position errors of the six points meet the requirements of the pasture-pushing task. The robot successfully returns to the starting point after completing the task of multi-point navigation, achieving autonomous navigation of the robot.

## 1. Introduction

With the development of agricultural and animal husbandry informatization, both the deployment of intelligent IoT devices and the placement of robot platforms have laid the foundation for the popularization of agricultural and animal husbandry modernization and become a research hotspot in the digital and intelligent agricultural and animal husbandry fields [1,2,3,4]. In order to solve the problems of labor intensity, low time efficiency, and feed waste during manual feed pushing in the pasture, nowadays, many pastures have introduced intelligent pushing robots [5,6,7,8], and in order to achieve accurate pushing, map construction is the top priority, which is a prerequisite for a mobile robot to achieve autonomous navigation. The core technology of map construction is Simultaneous Localization and Mapping (SLAM). SLAM technology can be very effective in constructing a grid map of the agricultural and animal husbandry areas; as the basis of action of a functional robot, whether it is pushing materials, delivering materials, or carrying out other actions, an accurate map is a prerequisite. Autonomous navigation technology is at the core of pasture-pushing robots, and autonomous navigation technology is at the core of overgrowth robots. However, existing navigation systems such as guide rails, magnetic stripe guidance, or visual navigation methods are difficult to apply to the complex cattle field environment; therefore, further research is needed to develop a navigation system suitable for pasture-pushing robots.

At present, the methods for the navigation and localization of pasture-pushing robots are mainly divided into two categories: external information-assisted navigation and autonomous exploration-based navigation. Based on external information-assisted navigation, it is difficult to meet the needs of today’s pasture pushing [9]. Autonomous exploration navigation refers to a navigation method in which the robot relies on its own sensors and algorithms to achieve environment sensing, map construction, and path planning without external guidance information [10]. The commonly used sensors include LiDAR, depth cameras, and IMUs (Inertial Measurement Units). Liu, K. et al. [11] designed an autonomous navigation system based on laser SLAM for a greenhouse tomato-picking robot for the specific operation’s path-cruising problem and verified the validity of laser SLAM for outdoor map building. Song, H. et al. [12] developed an autonomous navigation method to achieve autonomous navigation in a robot to complete the task of overthrowing forage in a cattle ranch environment based on simultaneous localization and map building with LiDAR; secondly, the obstacle avoidance strategy of the inspection robot in the complex environment of the ranch was explored, including obstacle detection, path planning, and dynamic obstacle avoidance; and lastly, the validity and practicability of the proposed technology were verified through experiments, which proves the important role of automatic navigation and obstacle avoidance technology of the laser SLAM-based forage-flipping robot in improving the operational efficiency and intelligence level of the pasture. However, the presence of numerous similar structures in the pasture environment, such as repeated fences and feed troughs, makes it difficult for LiDAR to extract sufficient feature points, thereby affecting the accuracy of pose estimation. Relying solely on LiDAR as the mapping sensor cannot meet the requirements for mapping in complex pasture environments.

In recent years, advancements in computer vision and the evolution of vision components have spurred extensive research into visual navigation algorithms. Zhou, X. et al. [13] proposed a pick point localization algorithm for grape key structures by integrating deep learning and multi-target recognition algorithms. They enhanced the localization of grape-harvesting positions by improving the YOLACT++ model to detect grape structures, thereby reducing damage and failure rates during the harvesting process. Tian, F. et al. [14] proposed an autonomous navigation method for a farm pushing robot based on machine vision. After capturing the environmental image with an RGB camera, they performed preprocessing steps including sky removal, denoising, and grayscale transformation. Subsequently, key features such as the front fence, feed belt, and ground were identified through image segmentation, and the edge of the feed belt was extracted as the navigation path. The study innovatively applied the particle swarm optimization algorithm to tune the PID control parameters, enabling the robot to maintain lateral deviations within ±15 cm (maximum deviation 8.9 cm, average 7.6 cm) under various initial postures, thereby meeting the requirements for cow feeding operations. Experiments demonstrated that this method significantly reduced dependence on external markers while ensuring navigation accuracy. However, it exhibited limitations in light sensitivity and insufficient dynamic livestock management capabilities. Dong, R. et al. [15] proposed an improved ORB-SLAM2 visual navigation method. By innovatively fusing multimodal information such as 3D point clouds, 2D occupancy features, and visual road signs, they constructed a multilevel map representation that includes localization, planning, and interaction layers. This approach effectively overcomes the inherent defects of the traditional ORB-SLAM2, such as insufficient information of sparse maps, lack of occupancy information, and poor reusability. Machine-vision navigation has many advantages such as accurate localization and fast recognition speed. However, visual navigation can only perform local planning, not global navigation planning, and it is easily affected by lighting conditions in outdoor environments [16]. The pasture environment is characterized by complex and variable lighting conditions, irregular spatial layouts, and diffused forage dust, among other factors. These unique working conditions highlight the limitations of single-sensor-based SLAM technologies: visual sensors are unstable under strong backlighting or at night, LiDAR is susceptible to interference from forage dust, such interference can distort point cloud data, and Inertial Measurement Units (IMUs) alone are prone to significant cumulative drift. Therefore, multi-sensor information fusion has emerged as a crucial technological approach to address the autonomous navigation challenges of pasture-pushing robots. Han, W. et al. [17] proposed a navigation system for autonomous positioning navigation in pepper fields, which is based on multi-sensors and an optimized TEB (Timed Elastic Band) algorithm. The system employs an extended Kalman filter to fuse data from LiDAR, IMU, and GNSS, thereby optimizing the TEB algorithm to enhance the navigation performance. Liu, M. et al. [18] proposed a SLAM optimization method based on the tight coupling of multi-line LiDAR and IMU. Their approach innovatively adopts a factor graph optimization framework, balances computational efficiency and accuracy through a sliding window mechanism, and ensures optimization quality by incorporating edge processing. Additionally, it integrates a scanning context closed-loop detection algorithm to improve the consistency of graph building. Experiments demonstrate that this method significantly enhances point–plane matching accuracy while maintaining real-time performance, providing a reliable localization and mapping solution for autonomous driving and mobile robots.

Wang, Z. et al. [19] proposed a novel fusion method to address the heterogeneity of LiDAR and depth cameras in terms of the measurement range, point cloud density, and distribution pattern. The scale difference is resolved using a moving sphere spatial coarse localization algorithm, while density inhomogeneity is managed by combining an improved FPFH (Fast Point Feature Histogram) coarse alignment with an enhanced ICP (Iterative Closest Point) fine-alignment algorithm. Experiments demonstrate that this method achieves a translation accuracy of 4.29 cm and a rotation accuracy of 0.006 rad when fusing a 64-wire LiDAR with a depth camera, effectively meeting the safety requirements for human–computer collaboration. Su, Z. et al. [20] proposed an autonomous navigation method for orchards based on multi-sensor fusion. Using a tracked agricultural platform as the test vehicle, they fused data from a 2D LiDAR, a dynamic electronic compass, and encoders to construct an autonomous orchard navigation system. Elamin, A. et al. [21] proposed an event-based visual inertial odometry approach that emphasizes adaptive event accumulation and selective keyframe updating to reduce computational overhead. The method integrates data from event cameras, visual odometry, and inertial measurements to achieve accurate indoor navigation. As discussed in the previous section, scholars have extensively researched three primary fusion schemes: laser–IMU, laser–camera, and camera–IMU. However, each of these schemes has notable limitations. LiDAR–IMU fusion is highly susceptible to single-feature influence, and its localization and mapping accuracy need improvement in long-distance roadway scenarios. Camera–IMU fusion SLAM fails in prolonged darkness and lacks full-scene application capability. The laser–camera fusion scheme also has significant limitations: in high-dust conditions typical of pastures, camera visibility is severely reduced, and LiDAR point clouds are significantly diminished due to dust scattering, resulting in substantial noise. These factors pose a significant risk of synergistic failure between the two sensors. Zhou, S. et al. [22] proposed an improved RTAB-Map–VIWO method to address the issue of accumulating localization errors in visual SLAM for indoor environments. Their method innovatively employs an EKF (extended Kalman filter) framework to fuse wheeled odometry and IMU data, providing optimized position predictions for RTAB-Map and effectively suppressing local error accumulation. Multi-sensor fusion experiments demonstrate that this method reduces RMSE by 48.1% compared to traditional RTAB-Map in public dataset tests. In real indoor scenarios, localization accuracy is improved by at least 29.4%, significantly enhancing the mobile robot’s trajectory tracking performance. Li, A. et al. [23] proposed a multi-sensor fusion SLAM and path-planning method for intelligent vehicles. By fusing LiDAR, depth camera, and IMU data through the EKF algorithm, they constructed a high-precision environment map. Combining an improved ant colony algorithm (for global path planning) with the dynamic window method (for local obstacle avoidance), they achieved path smoothing optimization and real-time obstacle avoidance. Experimental validation shows that this method significantly improves SLAM positioning accuracy and path-planning efficiency, providing an effective solution for unmanned systems to navigate autonomously in unknown environments. However, in the complex scenes of pastures, there is a scarcity of research on autonomous navigation algorithms based on multi-sensor fusion. The degree of fusion is low, often resembling a simple combination of laser–IMU and camera–IMU fusion schemes. The information fusion is insufficient to fully leverage the complementary advantages of multi-sensor data. To address the aforementioned challenges, this paper proposes an enhanced RTAB-Map algorithm based on multi-sensor fusion, aiming to refine the RTAB-Map methodology and integrate data from diverse sensors. By leveraging the strengths of various sensors, this approach yields more comprehensive, accurate, and reliable localization outcomes. The principal contributions of this paper are summarized as follows:

1. During map construction, Cartographer and RTAB-Map collaboratively share high-accuracy and robust fused trip information (TF). The RTAB-Map algorithm integrates data from the depth camera, LiDAR, and odometer to create a bimodal fused map, comprising a 2D raster map of the pasture and a 3D point cloud. This enhances the sensing capabilities of the pushing robot.

2. For navigation, we combine RTAB-Map’s global localization (low-frequency optimization) with AMCL’s local localization (high-frequency updating). By fusing IMU and odometer data through an EKF, we generate a smoother and more robust position estimate. We then optimize the navigation algorithm based on the fused data to achieve autonomous navigation of the pushing robot in the pasture environment.

3. The effectiveness of the proposed method is validated through experiments conducted in simulated pasture environments.

## 2. Related Work

### 2.1. Design of Navigation System for Pasture-Pushing Robot

#### 2.1.1. Operational Requirements for Research Context

**Figure 1:**
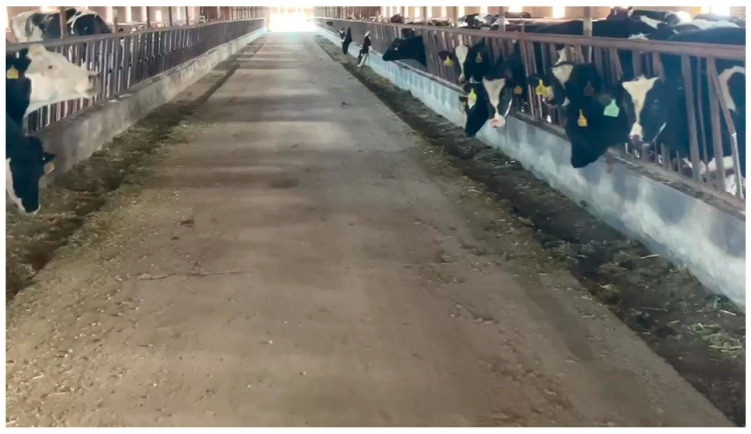
Pasture-pushing environment.

Figure 1 illustrates the actual pasture-pushing environment, where the majority of the pushing area is a long rectangle with clearly defined boundaries around the troughs. To navigate the complex scenes encountered during pasture material pushing, the robot needs to plan a rational route to ensure the accuracy of material distribution.

**Figure 2:**
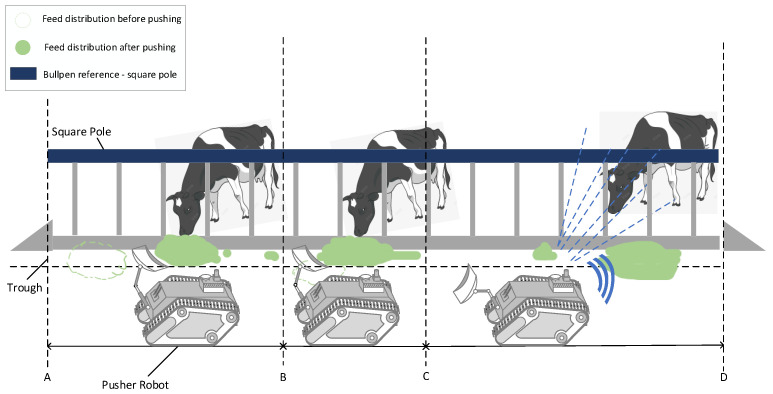
Pushing robot working principle. AB: Pushing Phase, BC: Completion Phase, CD: Recognition Phase.

Figure 2 illustrates the working principle of the pasture-pushing robot. The robot locates the feeding cows and those near the trough using the vision system. By integrating the laser SLAM algorithm, it senses the pasture environment information in real time and constructs the environment map. Through autonomous navigation technology, the robot avoids obstacles and pushes the forage back to the trough where the feeding cows are located.

**Figure 3:**
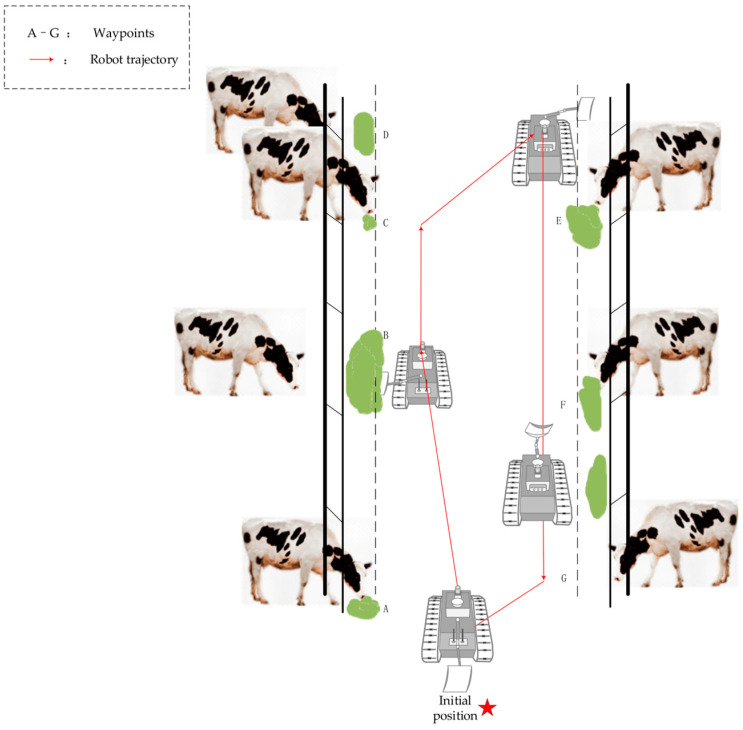
Schematic of robot path navigation.

Figure 3 illustrates the schematic of our robot’s navigation system, specifically designed for pasture environments that typically feature two side troughs and a central aisle. The robot’s primary function is to ensure a uniform distribution of feed across the feeding area, addressing any initial unevenness or displacement of feed from the troughs. After completing each feeding cycle, the robot returns to its designated starting point to await further instructions from a cloud-based control system. This setup enables centralized management and optimization of its operations.

Leveraging their practical knowledge, farm operators can set customized intervals for the robot to patrol the feeding areas, ensuring that the feed is evenly accessible to all cows. During each patrol, the robot updates its path points to refine the accuracy of its multi-point navigation system. This strategy, which integrates the operational insights of farm workers, offers a more practical and cost-effective solution compared to the less efficient method of continuous, round-the-clock operation. By aligning the robot’s activities with the natural feeding patterns and the expertise of the farm staff, we can significantly enhance the efficiency and uniformity of feed distribution, while also reducing operational costs.

**Figure 4:**
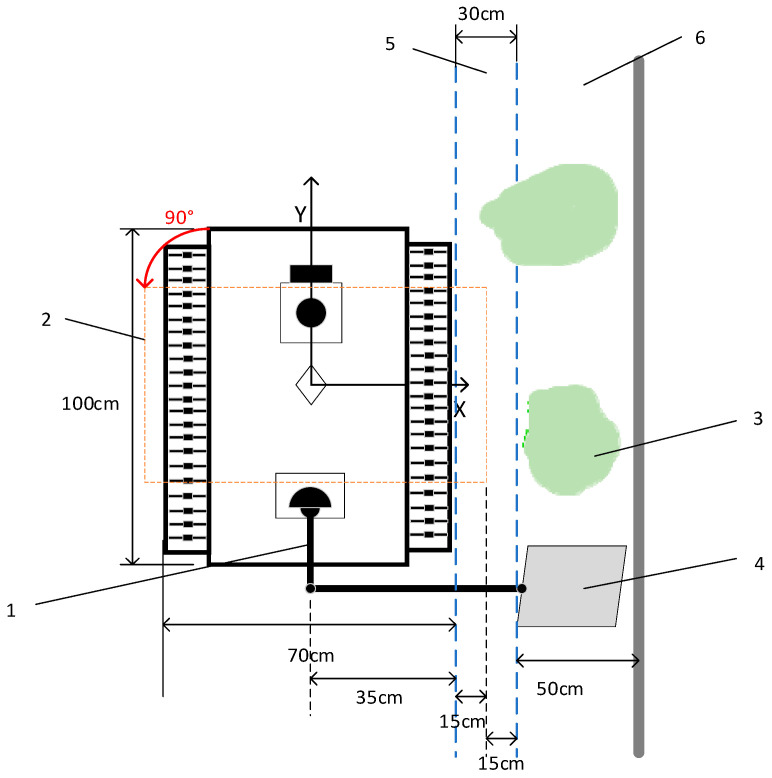
Schematic diagram of pushing operation area. Labels: 1, robotic arm; 2, robot after 90° turn; 3, forage; 4, pushing plate; 5, feed furthest arching distance; 6, chute.

Figure 4 illustrates the operation area of the pushing robot: the robot arm consists of two parts, with each joint having 360° mobility, ensuring the execution of various pushing operations. The pushing plate can be stowed when not in use. The robot measures 100 cm in length and 70 cm in width. Based on practical considerations, the robot is designed to reach up to 30 cm into the trough to ensure that cows can access the furthest feed. When the robot moves in a straight line, the distance between the robot and the trough can be adjusted in the X-direction, with a maximum distance of 30 cm. During a 90° turn at the maximum distance of 30 cm from the trough, the robot’s body will extend 15 cm beyond the furthest line of the trough. To avoid contact with cows during turns, the robot’s turning error must not exceed 15 cm. Additionally, to accurately push feed to the target cows in front of the robot, the maximum error in the Y-direction should not exceed 20 cm, considering the actual intervals of the pasture fence.

In summary, the navigation and localization requirements for the pasture-pushing robot are as follows:

Straight Line Walking: When the robot moves in a straight line along a 20-m corridor, the displacement in the *X*-axis direction must be kept within a maximum of 30 cm.

Inter-corridor Turning: When the robot turns and walks, the deviation from the turning path must not exceed 15 cm.

Multi-point Navigation and Fixed-point Parking: The robot updates the map at regular intervals. Each time, it determines the path points based on different feeding and cattle distribution information. The movement at the path points should be limited to a maximum of 30 cm in the X-direction and a maximum of 20 cm in the Y-direction.

#### 2.1.2. Robot Structure

**Figure 5:**
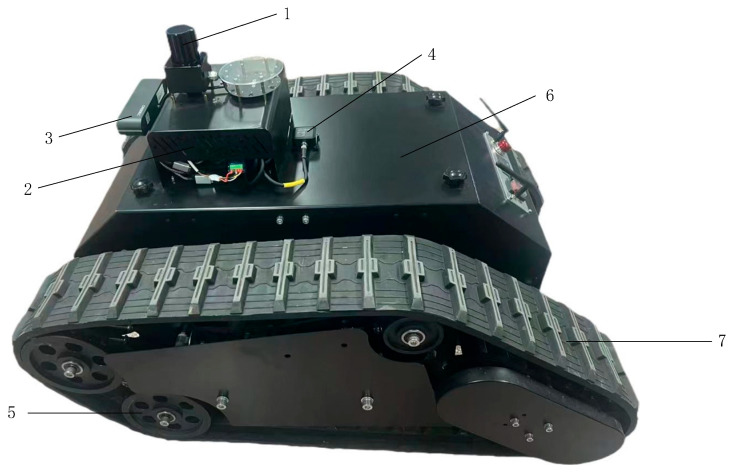
Ranch pushing robot architecture. Labels: 1, LiDAR; 2, ROS master; 3, depth camera; 4, IMU; 5, wheels; 6, sheel; 7, track.

Based on the above analysis and related parameters, we acquired a tracked vehicle, as shown in Figure 5. The vehicle measures 100 cm in length, 70 cm in width, and 50 cm in height. It is equipped with a LiDAR, a ROS master controller, a depth camera, an IMU, a housing, and a tracked walking structure. The functions, including map building and navigation, are achieved by embedding the improved algorithmic model into this robot. The LiDAR generates a 2D map of the ranch environment. The Jetson Nano in the ROS master controller runs various neural networks that perform functions such as image recognition, target detection, and localization. The tracked differential PID drive module enables 360° steering in place. The mechanical drive structure is designed to be suitable for a wide range of terrains. The enclosure protects the internal components.

**Figure 6:**
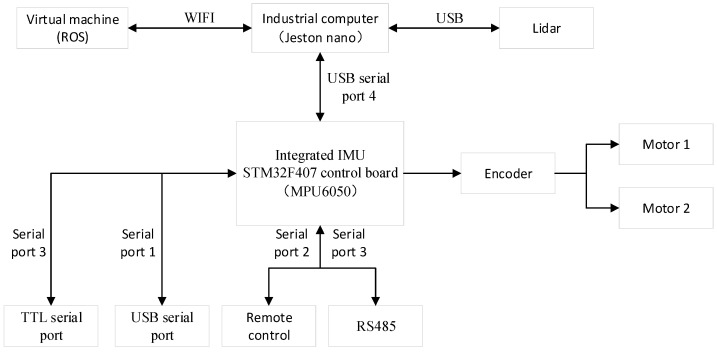
Pasture-pushing robot control system.

Figure 6 illustrates the control system of the pasture-pushing robot, which includes a virtual machine ROS system installed on an industrial computer, a LiDAR (LTME-02A, LIC, Shenzhen, China), a motion control unit (STM32F407, Shenlian Intelligent Equipment (Shenzhen) Co., China), and two brushless motors equipped (Shenlian Intelligent Equipment (Shenzhen) Co., China) with encoders. The robot’s main controller is an industrial computer running ROS (Shenlian Intelligent Equipment (Shenzhen) Co., China) on Ubuntu 18.04, communicating via WIFI, and accessible via a remote-control app (no machine). The industrial controller sends commands to the motion controller, which collects velocity feedback from the encoders, determines the robot’s motion attitude, extrapolates the trajectory to obtain odometry data, and transmits these data to the industrial computer via a USB serial port. The industrial computer integrates LiDAR observations with odometry data to generate a 2D grid map. Upon receiving a navigation command from the ROS system, the industrial computer plans the route and sends a command to the motion controller to drive the robot. The robot controls the brushless motors through a PID controller.

## 3. Experiment

### 3.1. Laser SLAM Algorithm Selection

#### Gazebo Emulation Platform Test

In the history of SLAM development, numerous excellent algorithms have emerged, primarily categorized into filtering methods and graph optimization methods. Among filtering methods, Gmapping stands out. It uses particle filtering as its core, improves the proposal distribution, optimizes the resampling strategy, and thereby enhances the algorithm’s reliability. The Hector algorithm, on the other hand, addresses the matching problem using the Gaussian–Newton method. Cartographer, a graph optimization-based SLAM algorithm, achieves efficient pose estimation and map construction by integrating local SLAM with global optimization. In local SLAM, sensor data are employed for scan matching to generate sub-maps and update positions. In global optimization, the factor graph serves as the basis, and loop closure detection corrects cumulative errors to ensure map consistency. Cartographer is known for its high accuracy and real-time performance, making it suitable for constructing 2D/3D raster maps in large-scale complex environments [24,25].

**Figure 7:**
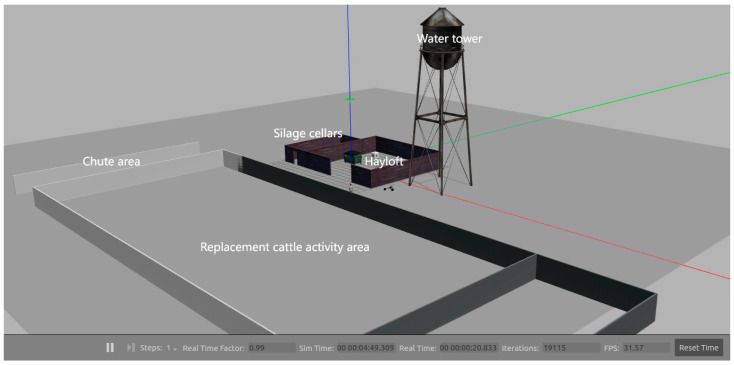
Simulation map.

To better utilize the LiDAR data, this paper constructs a simulated ranch environment in Gazebo based on the floor plan of Hohhot Shengqingyuan Agricultural and Animal Husbandry Co., Ltd., in Inner Mongolia, China. The simulation primarily focuses on the silage cellar, hay barn, and the activity area of the reserve cattle, including their trough area. The study investigates the advantages and limitations of three laser mapping schemes, as illustrated in Figure 7.

**Figure 8:**
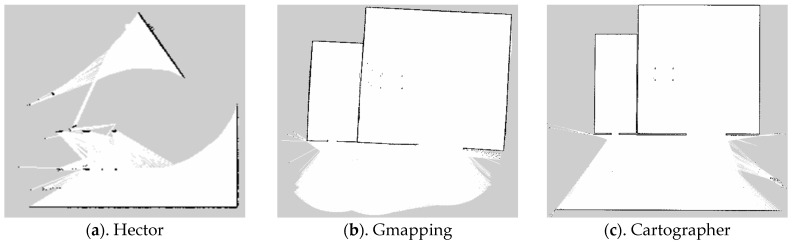
Built map effect.

**Figure 9:**
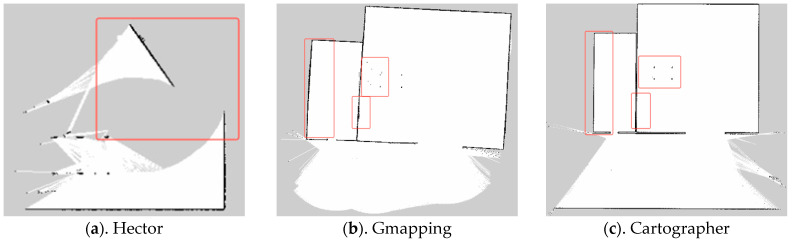
Effect comparison.

In ROS, the trolley was controlled via keyboard to navigate around the hay shed and silage cellar for one week, with the navigation package activated to ensure that the three algorithms—Hector, Gmapping, and Cartographer—operated under identical conditions. The map-building results of these algorithms are depicted in Figure 8. As indicated by the red rectangular box in Figure 9a, the Hector method exhibited significant drift during operation. Due to its frame-to-frame matching characteristic, map building and localization can be deemed unsuccessful when data from two consecutive frames fail to align with the recognition scan. This limitation restricts its applicability in outdoor mapping scenarios. In this experiment, neither Gmapping nor Cartographer exhibited large-scale drift at the same speed. However, Gmapping’s map detail reproduction was not as precise as Cartographer’s, with some straight lines appearing curved, as shown in the red boxes in Figure 9b,c. The map details were less refined compared to Cartographer, with some straight lines appearing curved, as illustrated in the red boxes in Figure 9c.

**Figure 10:**
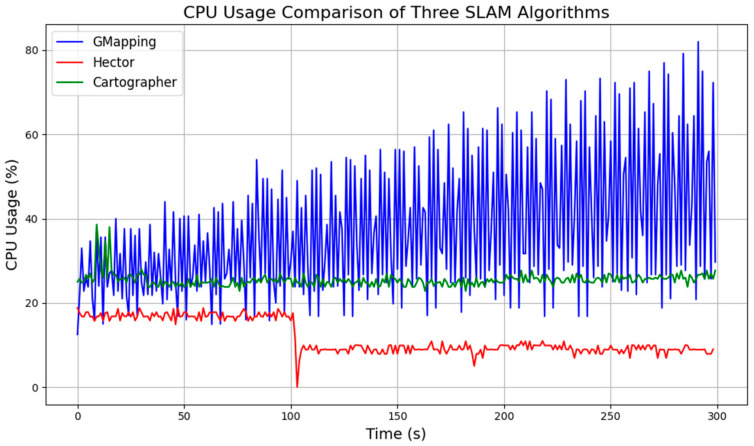
CPU usage comparison of three algorithms.

**Figure 11:**
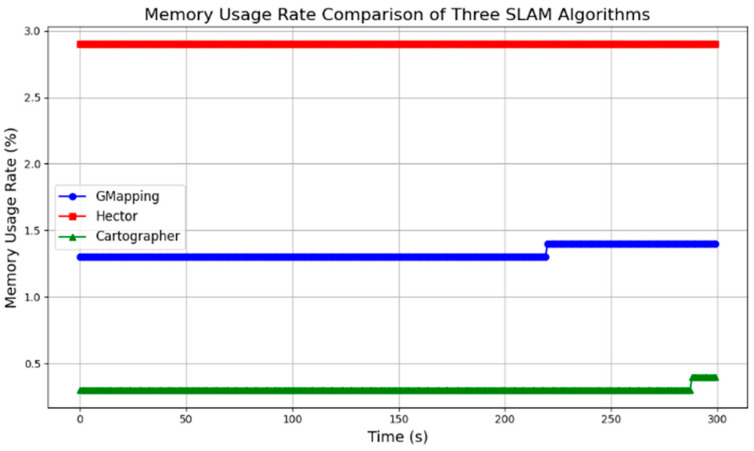
Memory usage rate comparison of three algorithms.

In addition, during the map-building process, there is a significant amount of data transfer between the robot’s master computer and the sensors. The algorithms place high demands on the computational power and memory of the computer. Therefore, it is necessary to test and compare the computational resource consumption of the algorithms. During the map-building process, the computer memory utilization and CPU occupancy were measured for each algorithm running separately. The results were plotted in the line graphs in Figure 10 and Figure 11.

Based on the data, Hector’s algorithm has the lowest CPU usage but the highest memory requirement. Gmapping’s memory requirement increases over time and has the highest CPU usage. Cartographer’s memory requirement is slightly lower than Gmapping’s, but its CPU occupancy rate is significantly higher than Gmapping’s.

Combining the above experimental results, the following analysis is made:

1. Hector’s algorithm demands the least computational power from the computer, However, its inter-frame matching strategy results in higher requirements for sensor accuracy and update frequency. According to the experiments, Hector may fail to at the robot’s traveling speed, at which Gmapping and Cartographer can successfully build the map.

2. Gmapping outperforms Hector in terms of robustness and accuracy. Nevertheless, due to the limitations of the particle filtering algorithm, Gmapping requires more computational resources.

3. Cartographer exhibits the highest robustness and accuracy among the three. Its back-end loop closure detection enhances algorithm performance. Additionally, the algorithm’s capability in incremental map building makes it well-suited for dynamic outdoor environments. Cartographer’s subgraph and global map optimization strategies conserve computational resources, rendering it less computationally intensive than Gmapping. Therefore, Cartographer’s algorithm is more suitable as a foundational method for outdoor map construction compared to the other two algorithms.

### 3.2. Mapping and Navigation Based on Multi-Sensor Fusion

#### 3.2.1. Principles and Improvements of the RTAB-Map Algorithm

RTAB-Map (real-time appearance-based mapping) is a real-time map-building algorithm based on appearance recognition. It is widely used in the fields of robot navigation, environment sensing, and 3D reconstruction. RTAB-Map recognizes the environment using visual features such as SIFT, SURF, or ORB, and constructs maps and performs localization by integrating data from multiple sensors. It supports data fusion from various sensors, including RGB-D cameras, LiDAR, and IMUs. RTAB-Map can generate both dense 3D point cloud maps and 2D raster maps. The algorithm optimizes map consistency through a loop closure detection mechanism. RTAB-Map is highly accurate, real-time, and flexible, making it suitable for dynamic environments and complex scenes.

**Figure 12:**
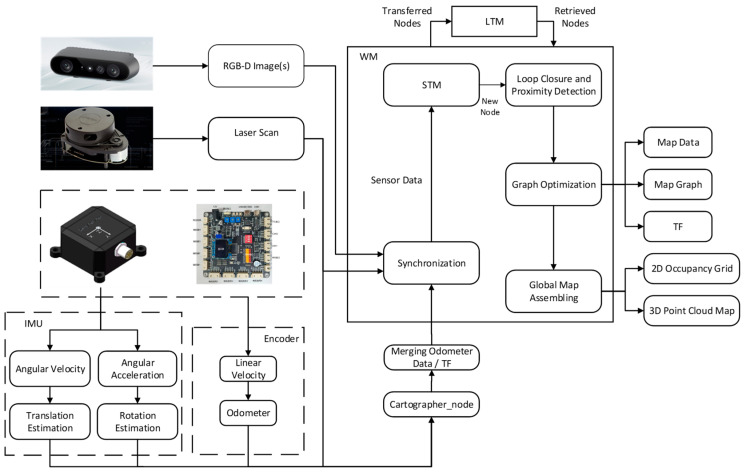
Improvement of RTAB-Map algorithm framework.

To meet the pasture-pushing robot’s requirement to sense its surrounding environment in real time during operation, this paper optimizes the RTAB-Map algorithm. By integrating data from the wheeled odometer, IMU sensor, and LiDAR, the algorithm enhances the robot’s position estimation accuracy and environment sensing ability during the pushing operation. During the pushing process, the wheeled odometer data provide short-term displacement change information, the IMU data assist in correcting attitude deviations, and the LiDAR scans the environment in real time to construct a 2D occupancy raster map and provide obstacle detection capabilities. The improved RTAB-Map algorithm enhances the robot’s environment sensing ability through multi-source data fusion, effectively reducing attitude drift caused by uneven ground and obstacles. This ensures the continuity and accuracy of the robot’s pushing operation. By leveraging Cartographer’s efficient map construction and optimization capabilities, the algorithm provides more accurate position estimation for RTAB-Map, thereby improving the accuracy of map construction and localization. This paper achieves the bimodal fusion of RTAB-Map and Cartographer for map building, ensuring that the pushing robot can quickly generate global maps and maintain accurate localization and path-planning capabilities in complex environments. The framework of the improved RTAB-Map algorithm is shown in Figure 12 [26].

**Figure 13:**
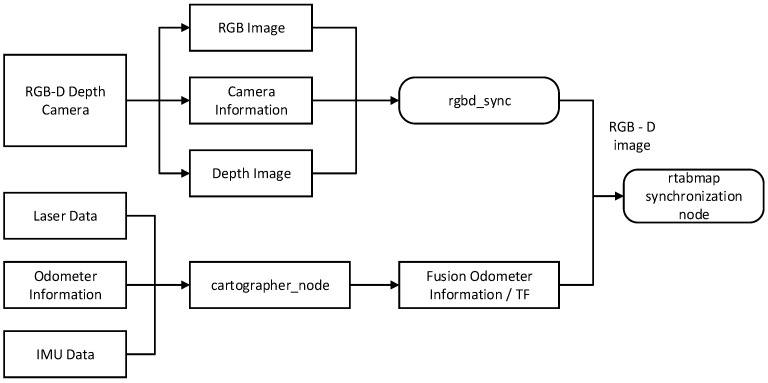
Sensor data synchronization process.

The input to the improved RTAB-Map algorithm comprises three main components: depth camera data, LiDAR data, and fused odometer data. The depth camera data are in RGB-D format, the LiDAR data are in Laserscan format, and the fused odometer data are fed into the system via a separate odometer node. The coordinates of the sensors and the robot’s chassis coordinates are input into the system through the Transform Frame (TF) software package (1.12.1). Since each type of data is input asynchronously through different topics, the synchronization module must first perform a timestamp alignment process. The data synchronization process is depicted in Figure 13. In this process, the rgbd_sync node accurately synchronizes the image and depth data from the RGB-D camera using the same timestamps. Subsequently, the system performs approximate synchronization with the LiDAR data and the fused odometer information to ensure the consistency of the multi-sensor data.

After completing synchronization, the sensor data are stored in the Short-Term Memory (STM) module, which creates corresponding map nodes for each frame of data and stores them. Simultaneously, all sensor data are transformed into the robot’s coordinate system using TF transformations based on LiDAR point cloud and depth camera data to generate localized 2D raster maps and 3D point cloud maps. Conversely, the odometer data provide position estimation information to ensure the accuracy of local map construction. Based on the odometer position information of each node, the system stitches the local maps together to form a global map. However, due to the cumulative error of the odometer, issues such as map drift or overlap may occur. Therefore, the accuracy of the odometer is crucial for the accuracy of the global map. The main improvement of this study is the highly robust fusion of LiDAR, odometer information, and IMU data using the Cartographer algorithm, which provides more accurate TF information to optimize the map-building process of RTAB-Map. Cartographer generates highly accurate robot position estimates from the optimized odometry and laser SLAM, and uses them as TF inputs to RTAB-Map. This enables RTAB-Map to build 3D point cloud maps based on more accurate position information, thereby reducing the impact of cumulative errors on map accuracy. Additionally, to further optimize the global map, closed-loop detection and global optimization are necessary. In the RTAB-Map algorithm, closed-loop detection is achieved using a visual bag-of-words model and a Bayesian filter. The visual bag-of-words model calculates the similarity between the current bit-pose node and the nodes in the working memory (WM), and updates the node’s weight based on this similarity to determine whether the node participates in closed-loop detection or is transferred to the Long-Term Memory (LTM) module.

(1) $$s\left(z_{t},z_{c}\right)=\left\{\begin{array}{c} \frac{N_{p}}{N_{z_{t}}},N_{z_{t}}\geq N_{z_{c}}\\ \frac{N_{p}}{N_{z_{c}}},N_{z_{t}}<N_{z_{c}} \end{array}\right.$$

In the formula, *Nz_t_* and *Nz_c_* represent the total number of words in the STM module at the current moment and the last moment, respectively, while *N_p_* is the number of word pair matches. Bayesian filtering is used to update the probability distribution of candidate node similarity and select the node most likely to trigger closed-loop detection by dynamically adjusting the probability distribution. Let the current bit-pose node be *L_t_*. All the candidate nodes to be detected in the working memory (WM) are considered as a whole and are represented by a random variable *S_t_*. The probability of *S_t_* = *i* represents the possibility of *L_t_* forming a closed loop with the candidate node *L_i_*. Based on Bayesian estimation, the updating process of this probability can be expressed as

(2) $$P\left(S_{t}\left|L^{t}\right.\right)=\eta \left(L_{t}|S_{t}\right)\sum_{i=1}^{t_{n}}P\left(S_{t}|S_{t-1}=i\right)P\left(S_{t-1}=i|L\right)$$

In the equation, *η* is the normalization constant; *L_t_* is the observation sequence, representing all the bit position nodes in the working memory (WM) at time *t*.

When a closed loop is successfully detected, all nodes and their constraint relationships are passed to the graph optimization module to perform global optimization and correct the global bit-position drift of the robot’s odometer. After optimization and adjustment, the local maps stored in each node can be further fused to construct a consistent global map, ultimately generating a global raster map suitable for navigation.

#### 3.2.2. Construction of a Multi-Sensor Fusion Navigation Algorithm Framework

**Figure 14:**
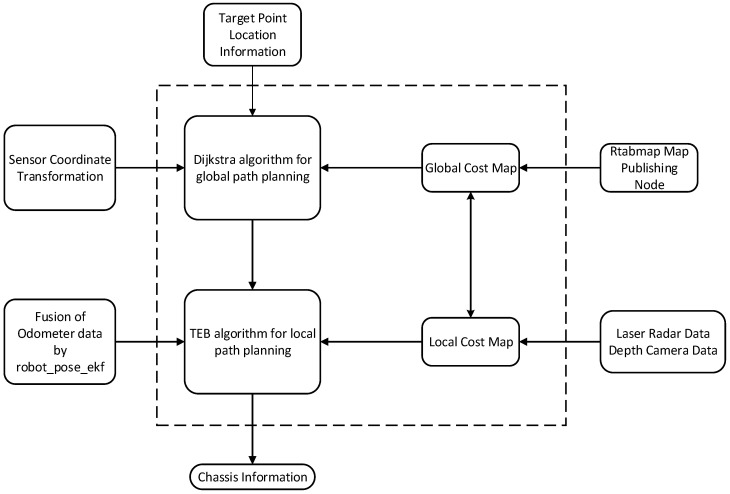
Navigation algorithm framework.

Path planning, robot localization, and autonomous obstacle avoidance are the three key components of autonomous robot navigation. In this study, efficient autonomous navigation of robots in complex environments is achieved by integrating multiple algorithms and techniques, with the algorithmic framework illustrated in Figure 14.

1. Localization method

In this study, the Adaptive Monte Carlo Localization (AMCL) algorithm is combined with the global localization capabilities of RTAB-Map to achieve precise robot positioning. AMCL, a probabilistic localization algorithm based on particle filtering, maintains a set of particles to represent possible robot positions and iteratively updates these distributions using sensor data and the motion model. This enables efficient real-time localization in dynamic environments with high robustness. RTAB-Map constructs highly accurate environment maps with global localization information by fusing multi-source data, such as depth camera, LiDAR, and odometer data. By integrating the local localization of AMCL with the global localization of RTAB-Map, the robot can maintain highly accurate position estimation in complex environments.

To further enhance localization accuracy and robustness, the extended Kalman filter (EKF) algorithm is introduced. The EKF fuses IMU and odometer data to generate smoother and more robust position estimates. IMU data provide acceleration and angular velocity information, while odometer data provide displacement information. By integrating these two data sources, the EKF effectively reduces single-sensor errors and improves position estimation accuracy, which is crucial for stable navigation in complex environments. Thus, by combining the global localization of RTAB-Map, the local localization of AMCL, and the fused position estimation of EKF, the robot achieves high-precision and high-robustness localization in complex environments.

2. Path-planning method

For path planning, this study adopts Dijkstra’s algorithm. Dijkstra’s algorithm is a classical single-source shortest path algorithm suitable for static environments. By modeling the ranch environment as a weighted graph, Dijkstra’s algorithm efficiently plans the optimal path from the starting point to the goal point. In dynamic environments, when combined with a multi-objective path optimization algorithm, Dijkstra’s algorithm can adjust the path in real time to adapt to environmental changes, ensuring the robot completes its tasks efficiently.

3. Autonomous navigation and obstacle avoidance

To address obstacles in dynamic environments, this study introduces the Temporal Elastic Band (TEB) algorithm. The TEB algorithm is an efficient local path-planning method, particularly well-suited for dynamic environments. By optimizing the temporal and spatial distribution of paths, the TEB algorithm can plan smooth and dynamic obstacle avoidance trajectories for the robot. It considers obstacle constraints, the robot’s motion model, and time costs to generate feasible paths that meet practical requirements. This enables the robot to efficiently complete tasks in dynamic environments (e.g., random movement of livestock or equipment) while reducing path length and travel time.

By integrating RTAB-Map for global localization, AMCL for local localization, and robot_pose_ekf for fused position estimation, this study significantly enhances the robot’s localization accuracy and robustness. The introduction of Dijkstra’s algorithm and the TEB algorithm further optimizes path planning and dynamic obstacle avoidance capabilities. The combined application of these methods enables the robot to achieve efficient and stable autonomous navigation in complex environments.

### 3.3. Autonomous Navigation Experiment in a Simulated Pasture Scenario

#### 3.3.1. Simulation Scenario Construction

**Figure 15:**
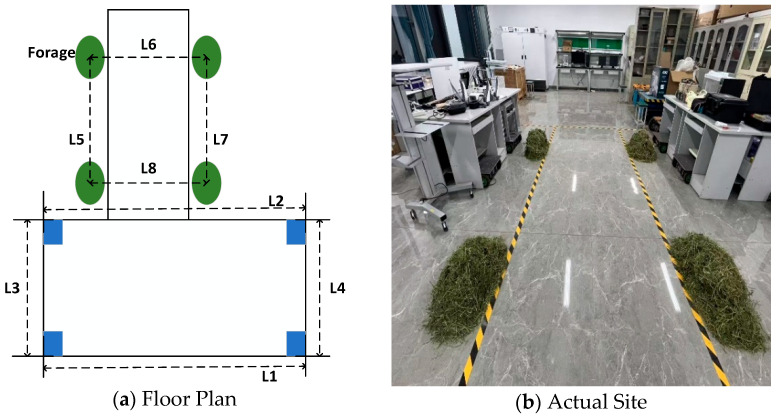
Experimental site.

During the autonomous navigation process, most pasture-pushing robots typically follow a rectangular trajectory to traverse the entire pasture environment. Prior to conducting field tests in the pasture environment, the overall performance of the robot’s autonomous navigation was assessed in a relatively ideal environment through a simulated path navigation experiment conducted in the Key Laboratory of Intelligent Perception and System Engineering of the Inner Mongolia Autonomous Region. The floor plan and the actual site are illustrated in Figure 15.

The dimensions of each location of the experimental site are shown in Table 1.

#### 3.3.2. Experimental Environmental Reconstruction of RTAB-Map Algorithm Incorporating Multi-Source Odometry Data

**Figure 16:**
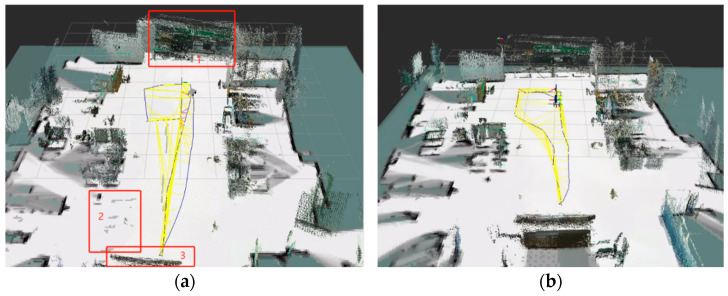
Comparison of 3D environment reconstruction: (**a**) pre-fusion and (**b**) post-fusion.

To evaluate the impact of the improved RTAB-Map algorithm on the reconstruction of the 3D environment, the algorithm was tested both before and after the fusion of multi-source odometry data in the simulated ranch scenario. The results are presented in Figure 16.

**Figure 17:**
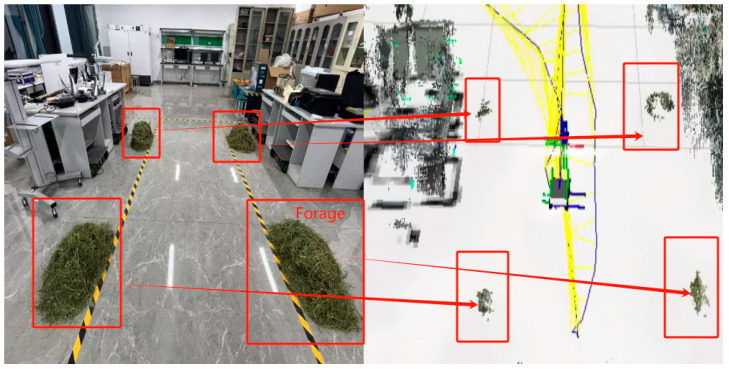
Laboratory mapping results.

Figure 16a illustrates the map construction effect before fusion, while Figure 16b shows the map construction effect after fusion. The environment map constructed by the RTAB-Map algorithm before optimization exhibits misalignments at locations 1 and 3 due to the discrepancies between the camera depth information and the laser point cloud data. At location 2, gray-white shadows and incomplete map construction are observed. However, these issues are effectively suppressed after optimization. The details of the constructed maps after fusion are shown in Figure 17.

The forage morphology in the laboratory scenario is accurately represented in the 3D point cloud map, with each position’s forage corresponding appropriately. However, due to limitations in camera placement and the robot’s movement range, the description of the small objects on the ground and objects in more distant positions is not perfect enough, Despite these limitations, the constructed maps are sufficient for the robot to perceive obstacles within its operational space.

To assess the accuracy of the RTAB-Map algorithm in reconstructing the environment before and after fusing multi-source odometry data, the dimensions of the reconstructed environment at eight specific locations (as shown in Figure 15) were compared with the actual dimensions. The absolute error and the relative error were calculated accordingly.

**Figure 18:**
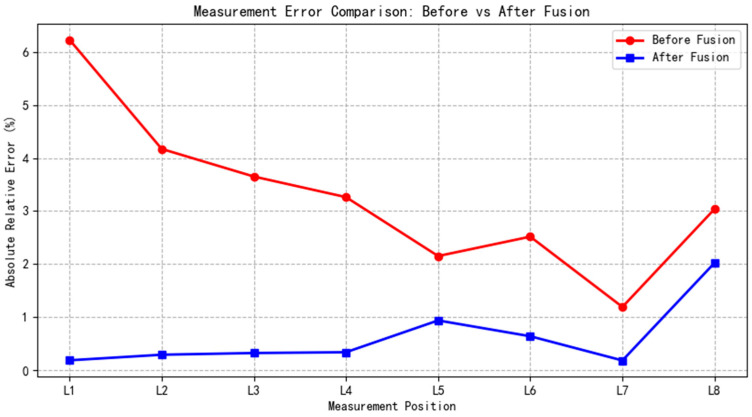
Comparative analysis of absolute errors before and after data fusion.

The results of the environment reconstruction dimensions for the eight specific locations, both before and after optimization, are presented in Table 2 and Table 3, respectively. Figure 18 illustrates a line graph comparing the relative error values.

By comparing the data in Table 2 and Table 3, it is evident that the maximum absolute error in map measurement size is reduced from 24.908 cm to 4.456 cm after fusion. The absolute error at each location is significantly decreased, and the maximum relative error is reduced from 6.227% to 2.025%. As shown in Figure 18, the relative errors after fusion are lower than those before fusion at each location. These results indicate that the integration of multi-source odometry data significantly enhances the performance of the RTAB-Map algorithm in the simulated ranch scenario, effectively mitigating large-scale offsets or drifts in the map construction process. The use of multi-source odometry fusion thus markedly improves the environmental reconstruction accuracy of the RTAB-Map algorithm in complex simulated ranch scenarios.

#### 3.3.3. Autonomous Navigation Experiment 1

**Figure 19:**
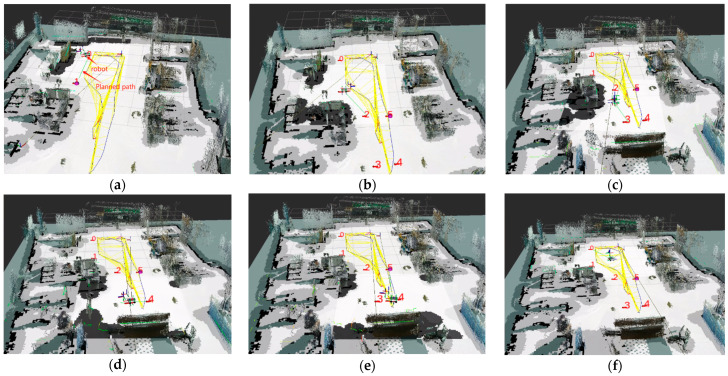
Laboratory navigation experiment. Points 0 through 5 are six navigation target points predefined in Rviz. (**a**) The process of robot navigation from Point 0 to Point 1. (**b**) The process of robot navigation from Point 1 to Point 2. (**c**) The process of robot navigation from Point 2 to Point 3. (**d**) The process of robot navigation from Point 3 to Point 4. (**e**) The process of robot navigation from Point 4 to Point 5. (**f**) The process of robot navigation from Point 5 to Point 0.

To verify the effectiveness of the robot’s autonomous navigation algorithm, a multi-point autonomous navigation test was conducted. The specific experimental process is as follows: Starting from the upper right stool (designated as point 0), the robot navigated to path points 1, 2, 3, 4, and 5 in a counterclockwise direction. Points 2, 3, 4, and 5 simulated the target locations for forage pushing. The navigation experiment is illustrated in Figure 19.

The Dijkstra algorithm was used to generate paths between points in the cost map. The Teb algorithm, based on odometry information provided by Cartographer, determined the robot’s trajectory and movement strategy, controlling the robot to autonomously navigate to the target positions and smoothly traverse the path points to complete the navigation task.

**Figure 20:**
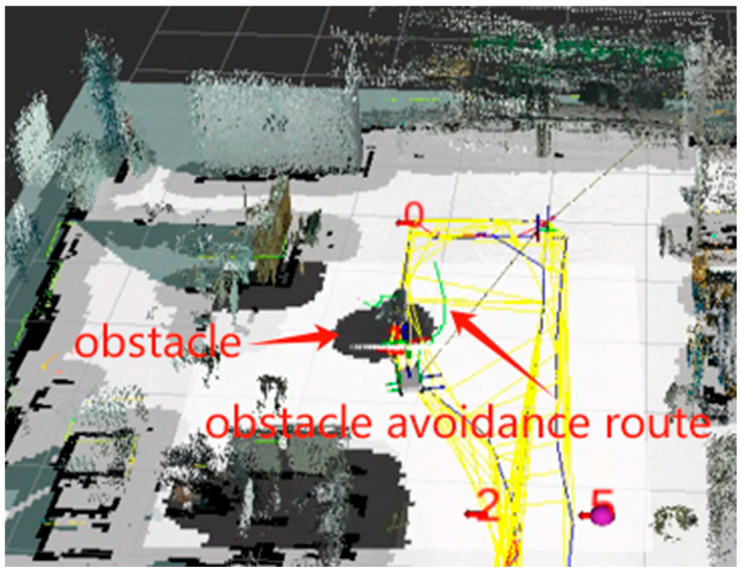
Obstacle avoidance experiment. Points 0, 1, 2, and 5 are four navigation target points predefined in Rviz.

Figure 19a–f illustrate the sequential traversal process, with the robot returning to point 0 after visiting each point. During this process, the robot effectively plans its path and smoothly avoids obstacles to reach the set target points, as shown in Figure 20. Additionally, the robot completes path planning and initiates autonomous navigation with a delay of less than 2 s, meeting the low-latency requirement.

The coordinates of the six navigation points were set using rviz and compared with the actual coordinates reached during navigation, as shown in Table 4. Comparison of the target coordinates of the six navigation points with the actual coordinates reached during the navigation process reveals that the maximum error is 20 cm in the X direction, 15 cm in the Y direction, and the maximum overall distance error from the target position is 17.3 cm, with a minimum error of 2.9 cm. These results meet the navigation requirements outlined in Section 2.1.1.

The distance error refers to the Euclidean distance from the coordinates of the target point to the actual coordinates, calculated using the following formula:

(3) $$d\left(P,\;Q\right)=\sqrt{{{(x}_{2}-x_{1})}^{2}+{{(y}_{2}-y_{1})}^{2}}$$

where target point *P* = (*x*_1_, *y*_1_); actual point *Q* = (*x*_2_, *y*_2_).

In addition, we used the EVO tool to compare our experimental results. EVO allows users to visualize, analyze, and evaluate SLAM algorithms and trajectory data. It helps to make the evaluation process more intuitive and informative. The evaluation metrics consider the absolute positional error (APE) values for both rotations and translations. APE is a metric used to assess the overall consistency of trajectories in SLAM.

APE is based on the absolute relative positional error between two positions, defined as

(4) $$T_{err}=T_{est}\cdot T_{ref}^{-1}=\left[\begin{array}{cc} R_{err} & t_{err}\\ 0 & 1 \end{array}\right]$$

where translation error: ${\left\|t_{err}\right\|}_{2}$ (Euclidean distance); rotation error: $\theta_{err}={\left\|\log R_{err}\right\|}_{2}$ (angle). For easy understanding, this paper uses the root mean square error (*RMSE*) to evaluate accuracy, which is calculated as shown below:

(5) $$RMSE=\sqrt{\frac{1}{N}\sum_{i=1}^{N}{\left\|t_{err,i}\right\|}^{2}}$$

The relevant metrics of the experiments are shown in Table 5. In the simulated ranch environment, the *RMSE* of the improved RTAB-Map algorithm is reduced by 1.75% and the standard deviation (Std) by 2.7% compared to the RTAB-Map algorithm. It is 26.7% lower than the AMCL algorithm and 22.8% lower than the standard deviation of the AMCL algorithm.

**Figure 21:**
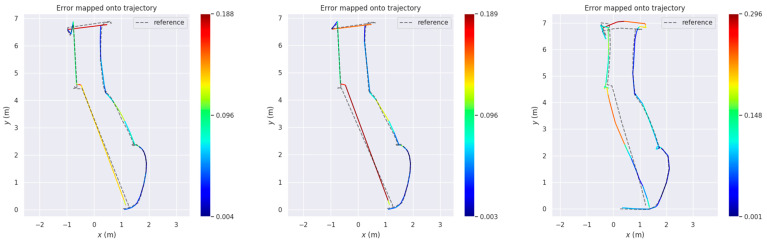

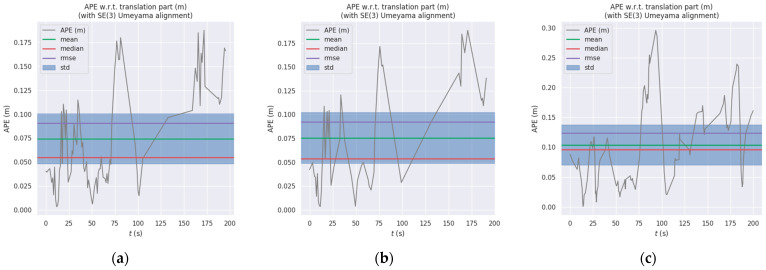
Absolute positional errors in Test 1 consider both rotational and translational errors. Column (**a**): Error trajectory of Improvemed_RTAB-Map and its APE over time. Column (**b**): Error trajectory of RTAB-Map and its APE over time. Column (**c**): Error trajectories of AMCL and their APEs over time. Reference: Actual trajectories of the robot.

**Figure 22:**
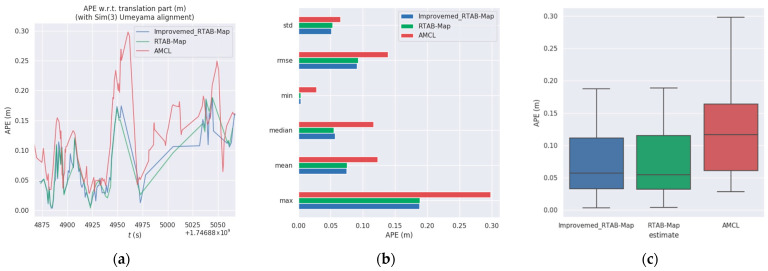
Comparison of APE values (RES) between Improvemed_RTAB-Map, RTAB-MAP, and AMCL in Test 1. Column (**a**) is a comparison of multiple APEs over time. Column (**b**) is a bar chart comparison. Column (**c**) is a box plot comparison.

The absolute positional error plots for the three algorithms are shown in Figure 21a–c. As seen in Figure 21 and Figure 22, the improved RTAB-Map algorithm performs the best in terms of error control, with smaller errors and greater consistency with the reference trajectory. The original RTAB-Map algorithm is second, with slightly larger errors but still maintaining good consistency. The AMCL algorithm has the largest errors and the greatest deviation from the reference trajectory, indicating relatively poor performance. This is attributed to the rich visual information in the RTAB-Map algorithm, which provides detailed visual features such as edges, corner points, and textures, thereby enhancing localization accuracy and map detail.

#### 3.3.4. Autonomous Navigation Experiment 2

**Figure 23:**
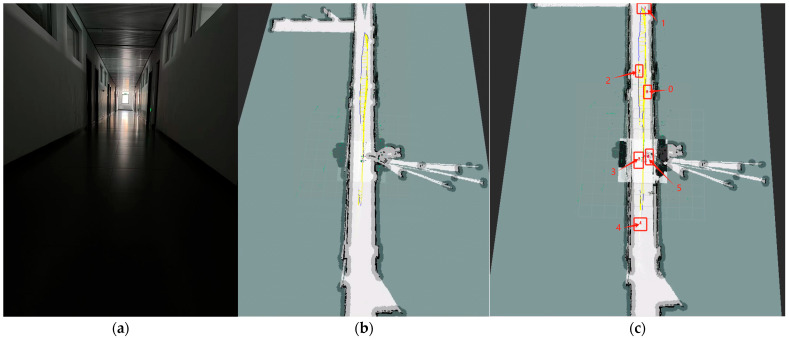
Experimental results from Test 2: (**a**) The actual testing environment (a 25 m × 2.1 m narrow corridor under overcast conditions). (**b**) The 3D map reconstructed using our proposed algorithm. (**c**) Multi-point navigation process with six designated waypoints. Points 0 through 5 are six navigation target points predefined in Rviz.

To validate the effectiveness of the improved algorithm under low-light conditions, a multi-point navigation experiment was conducted at six designated locations in Experimental Scenario 2. This scenario featured a narrow corridor measuring 25 m in length and 2.1 m in width, as illustrated in Figure 23. The experiments were performed under overcast weather conditions. Despite the dim lighting, the algorithm successfully generated high-quality 3D maps, as demonstrated in Figure 23b,c. Key metrics for Experiment 2 are summarized in Table 6.

In environments with poor illumination and limited physical features, the improved RTAB-Map algorithm achieved a 1.4% reduction in RMSE and a 0.8% decrease in Std compared to the original RTAB-Map. Although its RMSE and Std values were slightly higher than those of AMCL, it exhibited a 4.9% improvement in max error and an 18% reduction in median error.

**Figure 24:**
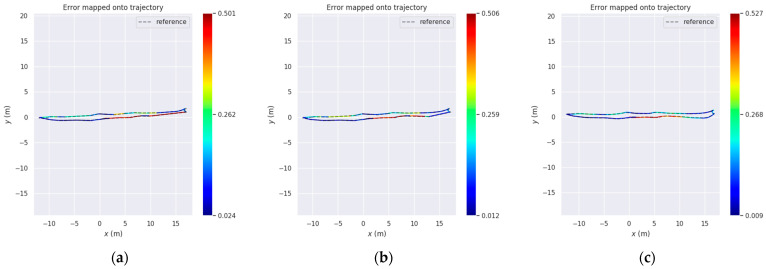
Absolute positional errors in Test 2 consider both rotational and translational errors. Column (**a**): Error trajectory of Improvemed_RTAB-Map. Column (**b**): Error trajectory of RTAB-Map. Column (**c**): Error trajectories of AMCL. Reference: actual trajectories of the robot.

Collectively analyzing the trajectory error maps (a, b, and c) in Figure 24, it is evident that the improved RTAB-Map algorithm generally performs the best, with the lowest average error and the least error fluctuation, particularly in the initial and middle segments of the trajectory. While the RTAB-Map algorithm is slightly less effective, its overall performance remains satisfactory. In contrast, the AMCL algorithm exhibits larger error fluctuations, especially in the latter part of the trajectory, indicating inferior stability and accuracy over extended periods compared to the other two algorithms. These observations suggest that the improved RTAB-Map algorithm offers significant advantages in terms of positioning accuracy and stability. However, further optimization is needed to address the high errors in specific areas.

**Figure 25:**
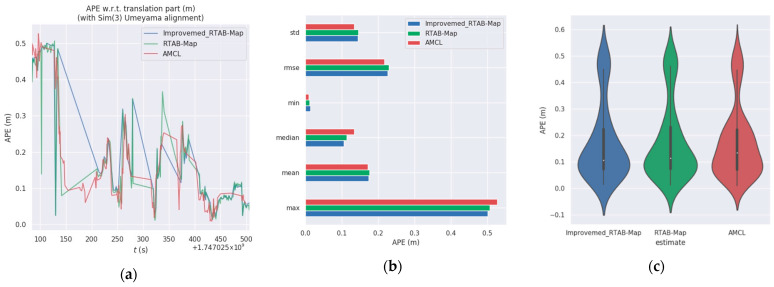
Comparison of APE values (RES) between Improvemed_RTAB-Map, RTAB-MAP, and AMCL in Test 2. Column (**a**) is a comparison of multiple APEs over time. Column (**b**) is a bar chart comparison. Column (**c**) is a comparison of violin plots.

From Figure 25a, it is evident that the improved RTAB-Map algorithm (blue curve) exhibits a lower absolute positional error (APE) than the RTAB-Map (green curve) and AMCL (red curve) for most of the time, indicating superior positioning accuracy in certain scenarios. However, the improved RTAB-Map algorithm shows significant fluctuations during specific time periods, such as between 200 and 250 s, suggesting potential stability issues during these intervals.

Figure 25b reveals that the improved RTAB-Map algorithm outperforms RTAB-Map and AMCL in most statistical indicators, particularly in terms of the maximum error, where the maximum error of the improved RTAB-Map algorithm is significantly lower than that of AMCL. However, the minimum error (min) of the improved RTAB-Map algorithm is very low, indicating that while the error can be minimal in some cases, it may increase significantly in others, reflecting potential issues with the algorithm’s adaptability and robustness in different environments.

Figure 25c shows that the distribution of the improved RTAB-Map algorithm is more concentrated with a lower median, indicating better positioning accuracy in most cases. However, the violin plot also reveals that the improved RTAB-Map algorithm may exhibit larger errors in some cases, suggesting limitations when dealing with extreme situations or outliers.

In summary, although the improved RTAB-Map algorithm generally outperforms other algorithms in most cases, it exhibits significant fluctuations and instability during certain periods and may have limitations when dealing with extreme situations. These limitations may be related to the algorithm’s parameter settings, environmental adaptability, or inherent robustness.

## 4. Results and Discussion

Firstly, in the RTAB-Map algorithm environment reconstruction experiment incorporating multi-source odometry, the improved RTAB-Map algorithm achieves a comprehensive description of the simulated ranch environment and the materials, thereby enhancing the accuracy of map construction. This experiment validates the effectiveness of the loosely coupled Cartographer–RTAB-Map architecture in map construction. Secondly, in the autonomous navigation experiment, based on the APE data in Table 5 and the comparison of error trajectories in Figure 21, it verifies the feasibility of combining global localization from RTAB-Map with local localization from AMCL to improve navigation and positioning accuracy. Finally, by integrating the results of the multipoint navigation and obstacle avoidance experiments, the navigation requirements outlined in Section 2.1.1 are satisfied.

In summary, leveraging the Cartographer–RTAB-Map loosely coupled architecture and the proposed multipoint navigation strategy, the study successfully implements multipoint autonomous navigation for the pasture-pushing robot, thereby enhancing work efficiency. Despite some limitations in stability and adaptability, the paper’s contributions lie in proposing an algorithm that offers higher precision and better stability under most circumstances. These achievements lay a valuable foundation for future research and applications while also highlighting areas for further study and optimization to achieve broader application and superior performance.

However, there are limitations to this work: Firstly, the robot with a tracked structure generates significant load on the tracks when operating on surfaces with a high friction coefficient, potentially leading to track derailment. Secondly, due to the physical limitations of the sensors, the 3D information captured in open scenes is less detailed compared to complex scenes, resulting in a lower degree of environment reconstruction.

## 5. Conclusions

To achieve the autonomous navigation of robots in the complex environment of pastures, this paper proposes an improved RTAB-Map algorithm based on multi-sensor fusion. For map construction, the cumulative error problem in long-time operation is addressed by sharing the Cartographer fused position estimation with the RTAB-Map algorithm. RTAB-Map then fuses multi-source data to construct a 3D point cloud map. By subscribing to the 2D map from the Cartographer algorithm and the 3D point cloud map from the RTAB-Map algorithm, the fused map is finally presented in Rviz. Experimental results demonstrate that the algorithm significantly improves positioning accuracy and map consistency while maintaining high real-time performance in the simulated ranch scenario. It effectively reduces the localization error and suppresses the offset or drift phenomenon due to accumulated error. For autonomous navigation, the robot provides the fused odometry (odom_combined) from the EKF algorithm to both the RTAB-Map and AMCL algorithms to achieve self-localization. The Dijkstra algorithm is used for shortest-path planning, and the Teb algorithm provides real-time obstacle avoidance for the robot. Experimental results show that the robot can efficiently accomplish multi-point navigation tasks while ensuring low latency. Therefore, this study is useful in promoting the three-dimensional mapping, autonomous navigation, and localization technology of pasture-pushing robots. It serves as a reference for other robots that require navigation and localization, offering high practical application value and development prospects. In the future, the specific realization of the pushing operation will be studied in depth and applied in practical scenarios as soon as possible.

## Figures and Tables

**Table 1:** Actual site dimensions.

Position	Measured Value/cm
L1	400
L2	400
L3	320
L4	320
L5	250
L6	220
L7	250
L8	220

**Table 2:** RTAB-Map algorithm dimensional measurements before fusion.

Position	Environmental Real Values /cm	Map Measurements/cm	Error Absolute Value /cm	Absolute Relative Error /%
L1	400	375.092	24.908	6.227
L2	400	383.315	16.685	4.171
L3	320	308.318	11.682	3.651
L4	320	309.559	10.441	3.263
L5	250	255.383	5.383	2.153
L6	220	225.539	5.539	2.518
L7	250	247.014	2.986	1.194
L8	220	226.689	6.689	3.040

**Table 3:** Fused RTAB-Map algorithm dimensional measurements.

Position	Environmental Real Values /cm	Map Measurements/cm	Error Absolute Value /cm	Absolute Relative Error /%
L1	400	400.756	0.756	0.189
L2	400	401.175	1.175	0.294
L3	320	321.042	1.042	0.326
L4	320	321.090	1.090	0.341
L5	250	252.351	2.351	0.940
L6	220	218.588	1.412	0.642
L7	250	250.466	0.466	0.186
L8	220	224.456	4.456	2.025

**Table 4:** Laboratory autonomy to navigate experimental information.

Position	Target Point Coordinate	Actual Coordinate	X Direction Error/m	Y Direction Error/m	Distance Error /m
0	(1.340, 0.147)	(1.349, 0.117)	0.009	0.030	0.031
1	(1.260, 2.270)	(1.370, 2.278)	0.110	0.080	0.094
2	(0.243, 4.400)	(0.259, 4.376)	0.016	0.024	0.029
3	(0.112, 6.880)	(0.312, 7.030)	0.200	0.150	0.173
4	(−0.877, 6.660)	(−1.007, 6.805)	0.130	0.145	0.137
5	(−0.667, 4.320)	(−0.546, 4.418)	0.121	0.098	0.109

**Table 5:** In Test 1, the APE values between our proposed method and RTAB-MAP as well as AMCL were compared.

Test-ID	Method	Std	RMSE	Min	Median	Mean	Max
Test 1	Improvemed RTAB-Map	0.051921	0.090829	0.003545	0.054863	0.074526	0.187753
	RTAB-Map	0.053364	0.092448	0.003425	0.053719	0.075491	0.188597
	AMCL	0.067224	0.123937	0.000707	0.096345	0.104122	0.296027

**Table 6:** In Test 2, the APE values between Improvemed RTAB-Map and RTAB-MAP as well as AMCL were compared.

Test-ID	Method	Std	RMSE	Min	Median	Mean	Max
	Improvemed RTAB-Map	0.144401	0.226886	0.023805	0.110312	0.175002	0.500843
Test 2	RTAB-Map	0.145673	0.230114	0.012198	0.118120	0.178134	0.506492
	AMCL	0.132963	0.219043	0.021416	0.134551	0.174070	0.526699

## Data Availability

The data presented in this study are available on request from the corresponding author. The data are not publicly available due to ongoing study.
